# Organic Acids Modulate Systemic Metabolic Perturbation Caused by *Salmonella* Pullorum Challenge in Early-Stage Broilers

**DOI:** 10.3389/fphys.2019.01418

**Published:** 2019-11-15

**Authors:** Jing Wang, Dong Dai, Hai-jun Zhang, Shu-geng Wu, Yan-ming Han, Yuan-yuan Wu, Guang-hai Qi

**Affiliations:** ^1^Key Laboratory of Feed Biotechnology of Ministry of Agriculture, Feed Research Institute, Chinese Academy of Agricultural Sciences, Beijing, China; ^2^Trouw Nutrition R&D, Amersfoort, Netherlands

**Keywords:** organic acids, *Salmonella* Pullorum, broilers, metabolomics, growth performance

## Abstract

The objectives of this study were to determine the protective effects of organic acids (OA) in broilers exposed to *Salmonella* Pullorum challenge at early stage and to explore the potential benefits of OA by metabolomics analysis. The treatment groups included non-challenged, *S.* Pullorum-challenged, challenged group supplemented with virginiamycin, challenged group supplemented with OA in drinking water, challenged group supplemented with OA in feed, and challenged group supplemented with OA in combination in drinking water and feed. Results showed that early *Salmonella* challenge induced an acute systemic infection of broilers in the starter phase, followed by the grower phase without triggering clinical signs. OA supplementation promoted growth during the grower phase, and while OA in water contributed more, the positive effects of OA in combination were comparable to those of virginiamycin supplementation in challenged birds. Furthermore, OA could modulate the systemic metabolic perturbation caused by challenge as it alleviated stress responses mediated by steroid hormone, potentially attenuated antioxidant or immune defense, and modified intestinal microbiota metabolism. These results show a metabolic mechanism that may partly explain the potential benefits of OA in *Salmonella* challenged birds, and may contribute to the use of OA to control or reduce *S.* Pullorum infection in farm animals.

## Introduction

*Salmonella enterica* subspecies enterica serovar Gallinarum biovar Pullorum (*S.* Pullorum) is a poultry-specific pathogen of considerable economic importance in China and also other countries with a developing poultry industry (Wigley et al., 2001). In China between 2006 and 2012, *S.* Pullorum was the most commonly detected serovar in chickens and the pullorum disease infection rates were reported to be over 30% (Li et al., 2013; Gong et al., 2014). In addition to causing high mortality rates among young chicks, one of the features of *S.* Pullorum infection is that it persists for long periods in convalescent chicks in the absence of clinical disease (Wigley et al., 2001). The chronic and mild depressive effect on growth or health of birds induced by *S.* Pullorum infection is hardly to detect in practice, which presents a high risk with huge associated economic losses, especially under the inadequate hygienic conditions. Another crucial problem is that of how to overcome the potential risk of bacterial infection when limiting the use of antibiotics in feed additives and veterinary antimicrobials. This is particularly pertinent as the MOA (Ministry of Agriculture and Rural Affairs of the People’s Republic of China) has proposed a deadline of December 2020 to complete the exit plan of medicated additives in feed production. Simultaneously, the MOA has carried out a pilot program for reduction of veterinary antimicrobial use from 2018 to 2021 in ≥100 large-scale farms every year. Much attention has been given to an exploitation of various antibiotic alternatives, assessment of their efficiency and safety, and investigation of potentially physiological and biomedical mechanisms.

Organic acids (OA), which primarily composed of short-chain fatty acids (SCFAs, ≤C6) and medium-chain fatty acids, are both bacteriostatic and bactericidal, and thus show promise as antibiotic alternatives in reducing colonization by pathogens. A series of studies have tested the efficacy of OA on reducing colonization with *Salmonella* in broiler chickens, turkeys, and pigs in an agricultural setting, and controlling *Salmonella* in meat and poultry products post-slaughter (Mani-Lopez et al., 2012; Arguello et al., 2013; Evans et al., 2017; Bourassa et al., 2018). The primary mechanisms of OA for reducing pathogenic bacteria include cytoplasmic acidification with subsequent uncoupling of energy production and regulation, and the accumulation of the dissociated acid anion to toxic levels *in vitro* (Mani-Lopez et al., 2012). This activity *in vivo* is heavily dependent on the concentrations of OA achieved in the animal gut, diet composition and buffering within the diet, potential acid-resistance of *Salmonella*, and other factors (Suiryanrayna and Ramana, 2015). Furthermore, OA have been reported to benefit broiler and swine production by promoting growth, lowering the pH of the gastrointestinal tract, modifying the gastrointestinal tract and gut microbiota, enhancing immune responses, and other physiological functions (Suiryanrayna and Ramana, 2015; Khan and Iqbal, 2016; Dittoe et al., 2018; Hamid et al., 2018; Liu et al., 2018). However, it is still unclear how OA could efficiently prevent infection in the animal gut and promote growth. There is also very little information concerning the effects of OA on physiological responses of infected broiler chickens, i.e., the role of OA on *Salmonella*-host interaction based on metabolic levels.

A wide range of OA with variable physical and chemical properties exists, of which many are used as drinking water supplements or as feed additives. Two commercial products tested previously in broilers and pigs were used in the current study (Chaveerach et al., 2002; Abudabos et al., 2014; Liu et al., 2018). The objective of this study was initially to determine the protective effects of OA in the feed and/or water against subclinical infection of *S.* Pullorum in broilers at early stages. Subsequently, we applied metabolomics to characterize metabolite changes due to *S.* Pullorum challenge and OA intervention. Our findings may inform on the potential benefits of OA and further develop a promising intervention strategy for employing OA to control and reduce *S.* Pullorum in livestock.

## Materials and Methods

### Animal Experiments

All experimental protocols were approved by the Animal Care and Use Committee of the Feed Research Institute of the Chinese Academy of Agricultural Sciences. A total of 672 newly hatched male Arbor Acre broiler chicks were randomly allocated into one of the six experimental feeding treatments. The treatments included: non-challenged group supplied with the control basal diets; *S.* Pullorum-challenged group with the basal diets (challenged control); challenged group receiving the control diets supplemented with 0.002% virginiamycin (feed grade premix, 50%; Phibro Animal Health Co., Ltd.) (challenge + virginiamycin); challenged group receiving the control diets and the drinking water added with OA blend (challenge + OA water); challenged group receiving the diets supplemented with OA feed additives (challenge + OA feed); challenged group receiving the combination of acidified drinking water and OA feed additives (challenge + OA water + OA feed). Each group consisted of eight replicates of 14 birds each. Diets were formulated to meet or exceed NRC (1994) guidelines (Supplementary Table S1), and both diets and water were supplied *ad libitum* in pellet form and by nipple drinkers, respectively. The experiment last for 6 weeks with body weight was recorded weekly during the starter phase (days 1 to 21) as well as at the end of trial (day 42). Feed intake was recorded weekly during all phase and mortality was monitored daily. Following this, the feed conversion ratio (FCR, feed:gain) and mortality were calculated.

### Materials

*Salmonella* Pullorum (CVCC521, C79-5) was obtained from China Institute of Veterinary Drug Control. The drinking water acidifier (Selko B.V., Netherlands) is a blend of free and buffered SCFAs, consisting of formic acid, acetic acid, and ammonium formate as active ingredients. The acidifier supplemented drinking water at around 1.5 L per 1000 L in the starter (0–21 days) and 1.0 L per 1000 L in the grower (22–42 days) period by using a separate waterline. The pH was reduced to 4.29 ± 0.21 in the acidified drinking water, while the rearing water pH on the farm level was range from 7.34 to 7.88 (Supplementary Table S2). The OA feed additives (Selko B.V., Netherlands) is a synergistic blend of OA (mainly formic acid, sorbic acid, propionic acid), MCFA (C8 to C12), butyrates, and hydrolyzed copra meal. The OA feed additives were added to the basal diets at 0.3% in the starter (0–21 days) and 0.1% in the grower (22–42 days) period. The combination of treated feed and water was at the same concentrations.

### *Salmonella* Infection

A pre-trial has been carried out to obtain the appropriate challenge does (10^9^ cfu/bird) of *Salmonella* Pullorum. On day 8 of age, birds of five challenge groups were infected with *Salmonella* Pullorum by oral gavage (1 × 10^9^ cfu/mL/bird) using an animal feeding needle for 3 consecutive days.

### *Salmonella* Count Analysis

On days 7, 14, 21, 35, and 42, one bird from each replicate, close to the average body weight, was euthanized by cervical dislocation. Then two sides of ceca were isolated and excised. The contents from one side of the ceca were weighed, placed into sterile plastic tubes, and diluted by 1:10 with 10-mL phosphate-buffered saline. The supernatant was obtained after centrifugation and serially diluted (serial 10-fold dilutions up to 10^7^). Black colonies were counted on *Salmonella-Shigella* Agar plates after incubation for 24 h at 37°C as previously reported (Agunos et al., 2007).

### Intestinal Morphological Analysis

On days 21 and 42, tissue samples were excised from the middle of jejunum and ileum for morphological measurement. Formalin-fixed samples were prepared using paraffin embedding procedures by sectioning 5 μm and stained with hematoxylin and eosin. A total of 15 intact, well-oriented crypt-villi units per sample were randomly selected and measured. The villus height (from the tip of the villus to the crypt opening) and crypt depth (from the base of the crypt to the level of the crypt opening) were determined using an image processing and analyzing system (NIKON CI-S and NIKON DS-U3, Tokyo, Japan).

### Metabolomic Profiling of Plasma

On day 42, one bird each replicate with average BW was selected from the non-challenged control, challenged control, and challenge + OA water group for blood sampling after a 12-h fast. Another two birds per group with average BW were selected to provide alternative blood samples. Blood samples were drawn from wing veins, and then centrifuged (3000 × *g* for 10 min) at 4°C to obtain plasma, before being stored at −80°C. Then, a total of 10 plasma samples were prepared before metabolomics analysis. Briefly, plasma samples were thawed at 4°C, and 40 μL of each sample was mixed with 120 μL cold methanol. The mixture was vortexed, ground, and centrifuged at 4000 × *g* for 30 min at 4°C. The supernatant (25 μL) was diluted to 1:19 with 50% methanol, and 10 μL each of diluted sample was placed in an injection vial to be assayed for metabolomic analysis.

The ultra-performance liquid chromatography (UPLC) analysis was performed using an Acquity UPLC system (Waters Corporation, Milford, MA, United States) equipped with a high-resolution tandem mass spectrometer (Xevo G2-XS QTOF, Waters MS Technologies, Manchester, United Kingdom). An ACQUITY UPLC BEH C18 column (2.1 mm × 100 mm, 1.7 μm, Waters MS Technologies, Manchester, United Kingdom) was used for the reversed phase separation. The column oven was maintained at 50°C. The flow rate was 0.4 mL/min and the mobile phase consisted of solvent A (water + 0.1% formic acid) and solvent B (acetonitrile + 0.1% formic acid). Gradient elution conditions were set as follows: 0–2 min, 100% phase A; 2–11 min, 0% to 100% B; 11–13 min, 100% B; 13∼15 min, 0–100% A. The injection volume for each sample was 10 μL. The Q-TOF was operated in both positive and negative ion modes. For the positive ion mode, the capillary and sampling cone voltages were set at 3.0 kV and 40.0 V, respectively. For the negative ion mode, the capillary and sampling cone voltages were set at 2.0 kV and 40.0 V, respectively. The mass spectrometry data were acquired in Centroid MSE mode. The TOF mass range was from 50 to 1200 Da and the scan time was 0.2 s. For the MS/MS detection, all precursors were fragmented using 20–40 eV, and the scan time was 0.2 s. During the acquisition, the LE signal was acquired every 3 s to calibrate the mass accuracy. Furthermore, in order to evaluate the reproducibility and stability of the LC-MS during the whole acquisition, a quality control sample (pool of all samples) was acquired after every 10 samples.

### Biochemical Measurements

Plasma levels of total protein, albumin, uric acid, and glucose were determined using an automatic biochemical analyzer (KHB300, Shanghai Kehua Bio-engineering Co., Ltd., Shanghai, China). Colorimetric kits (Nanjing Jiancheng Bioengineering Institute, Nanjing, China) were used to measure levels of glutathione and total anti-oxidative capacity (T-AOC) in the plasma samples. Each sample was analyzed in duplicate.

### Statistical Analysis

For metabolic profiling, the acquired raw data were processed with Progenesis QI software package (Progenesis QI Version 2.2, Non-linear Dynamics, Newcastle, United Kingdom) for alignment and filtration. Spectral deconvolution and normalization to the total ion amount generated a date matrix involving tR, *m*/*z*, and normalized peak area. The analytical variation was corrected with the quality control-based robust LOESS signal correction (QC-RLSC) algorithm. A threshold of 30% was set for the relative *SD* values of metabolites in the QC samples. All data were generalized logarithm-transformed and Pareto scaled before multivariate statistical analysis, which included an unsupervised principal component analysis (PCA) and partial least squares discriminant analysis (PLS-DA). The different metabolites were determined by the combination of the VIP value >1 of the PLS-DA model and the *P*-values (< 0.05) from Kruskal–Wallis test on the normalized peak intensities. Fold change was calculated as binary logarithm of average normalized peak intensity ratio between two groups. HMDB and KEGG databases were used to check and confirm the putative differential metabolites.

The other data were analyzed using the one-way analysis of variance (ANOVA), and means were compared using Duncan’s multiple range test (SPSS 19.0 for Windows, SPSS Inc., Chicago, IL, United States). The replicate was regarded as an experimental unit for analysis of performance data, and individual birds served as an experimental unit for the other parameters. Differences were considered statistically significant at *P* < 0.05, and the data are expressed as mean ± *SD*.

## Results

### General Health and Growth Performance

Significant differences in performance and mortality among groups were not observed during days 0–7 (*P* > 0.05), which suggested all chicks remained in good health during the pre-challenge period (Supplementary Table S3). All the challenged groups had decreased average daily feed intake compared to the unchallenged control during days 8–14 (*P* < 0.01). Lower average daily gain (ADG) and body weight on day 14 were also observed in challenged groups, with the exception of the group with dietary supplementation of virginiamycin, when compared to the unchallenged control (*P* < 0.01). Compared with the unchallenged group and virginiamycin group, the group receiving OA water, OA feed, or a combination thereof did not show any difference in performance during days 15–21 (*P* > 0.05). As shown in Table 1, the *S.* Pullorum challenge decreased ADG (*P* = 0.01), feed intake (*P* = 0.024), and body weight of birds compared to the unchallenged control during starter phase (days 1–21), and increased FCR during grower phase (days 22–42, *P* = 0.020). Higher ADG and body weight during grower (*P* = 0.009) and whole phase (*P* = 0.016) were observed in the challenged birds receiving the combination of OA drinking water and OA feed additives compared to the challenged control. The positive effect of combined feed and drinking water supplementation on performance was comparable to virginiamycin in challenged birds, evidenced by no difference between the virginiamycin and combination group during grower and whole phase (*P* > 0.05). In addition, the performance data during grower and whole phase showed that the challenged birds fed with OA feed additives had lower ADG and FCR than birds in the combination group. These data suggested that acidified drinking water may contribute more to the positive effects of the combination on performance.

**TABLE 1:** Effect of dietary supplementation with organic acids in drinking water or in feed on growth performance of broilers (0–42 days) exposed to *Salmonella* Pullorum challenge^d^.

**Items^e^**	**Unchallenge control**	**Challenge control**	**Challenge + VG^f^**	**Challenge + OA^g^ water**	**Challenge + OA^g^ feed**	**Challenge + OA^g^ water + OA^g^ feed**	***P*-value**
**Starter phase (0–21 days)**							
BW day 21 (g)	928.45 ± 34.81^a^	876.7 ± 42.25^b^	933.25 ± 41.88^a^	891.33 ± 28.93^ab^	901.1 ± 19.35^ab^	918.36 ± 29.5^ab^	0.027
ADG (g)	33.21 ± 2.17^ab^	30.88 ± 1.76^c^	33.85 ± 1.50^a^	31.29 ± 1.75^bc^	31.95 ± 1.12^abc^	32.09 ± 1.24^abc^	0.015
ADFI (g)	55.10 ± 2.04^a^	52.07 ± 2.56^b^	53.10 ± 1.66^ab^	51.92 ± 2.48^b^	51.43 ± 2.11^b^	52.7 ± 2.00^b^	0.024
FCR	1.64 ± 0.07^ab^	1.68 ± 0.06^a^	1.57 ± 0.03^c^	1.66 ± 0.05^ab^	1.62 ± 0.05^bc^	1.66 ± 0.01^ab^	0.012
Mortality (%)	0.00 ± 0.00	2.68 ± 3.70	0.89 ± 2.53	2.68 ± 3.70	2.68 ± 3.70	4.46 ± 5.31	0.213
**Grower phase (22–42 days)**							
BW day 42 (g)	2409.38 ± 126.01^ab^	2278.85 ± 106.51^b^	2506.99 ± 196.18^a^	2439.37 ± 83.87^a^	2371.38 ± 119.72^ab^	2501.99 ± 192.18^a^	0.026
ADG (g)	80.04 ± 3.40^b^	75.35 ± 2.58^b^	82.89 ± 9.85^ab^	81.72 ± 3.69^ab^	76.22 ± 7.86^b^	87.67 ± 5.22^a^	0.009
ADFI (g)	156.15 ± 6.85	153.41 ± 10.04	158.37 ± 13.27	157.96 ± 6.61	157.10 ± 13.83	168.70 ± 8.90	0.093
FCR	1.97 ± 0.05^b^	2.07 ± 0.16^a^	1.95 ± 0.09^b^	1.98 ± 0.07^ab^	2.08 ± 0.08^a^	1.94 ± 0.06^b^	0.020
Mortality (%)	4.55 ± 6.87	2.50 ± 4.63	4.66 ± 6.96	4.66 ± 6.96	7.27 ± 8.80	1.14 ± 3.21	0.610
**Whole phase (0–42 days)**							
ADG (g)	51.57 ± 3.07^ab^	49.09 ± 3.04^b^	53.98 ± 3.74^a^	51.11 ± 2.51^ab^	49.46 ± 3.37^b^	53.86 ± 3.12^a^	0.016
ADFI (g)	96.79 ± 3.72	92.15 ± 4.22	96.32 ± 4.76	95.72 ± 3.69	95.47 ± 5.12	99.23 ± 3.66	0.072
FCR	1.88 ± 0.08^ab^	1.90 ± 0.10^a^	1.80 ± 0.05^b^	1.87 ± 0.05^ab^	1.93 ± 0.06^a^	1.88 ± 0.09^ab^	0.048
Mortality (%)	3.57 ± 5.40	4.46 ± 6.54	4.46 ± 6.54	6.25 ± 5.96	8.04 ± 8.90	5.36 ± 5.05	0.792

*^*a*–*c*^Means within a row with no common superscript differ significantly (*n* = 8; *P* < 0.05). Data are the mean of eight replicates with 14 birds each. ^*d*^Birds were challenged with *S. enteric* subsp. Pullorum on day 8–10. The same as below. ^*e*^BW, body weight; ADG, average daily gain; ADFI, average daily feed intake; FCR, feed conversion ratio (feed:gain, g:g); ^*f*^VG, Virginiamycin feed grade premix; ^*g*^OA, organic acids.*

### Cecal Chyme *Salmonella* Count

As shown in Table 2, on day 7, there were no significant differences in cecal chyme *Salmonella* count among groups (*P* > 0.05), which is consistent with observed performance before *S*. Pullorum infection. On day 14 (post-infection), the amount of recovered *Salmonella* was significantly increased in challenged birds fed the control diet than unchallenged birds. But challenged birds receiving OA water, OA feed, or this supplementation in combination did not show any difference in recovered *Salmonella* compared with unchallenged or challenged birds (*P* > 0.05). It is interesting to find that lower amounts of *Salmonella* in challenged groups receiving OA water and the combination of OA water and OA feed additives than the challenged control on day 42 (*P* < 0.001). The virginiamycin group birds always displayed a reduced amount of recovered *Salmonella* from cecal chyme compared to the challenged control on days 14, 21, and 42 (*P* < 0.001), as well as the unchallenged group on days 21 and 42 (*P* < 0.001).

**TABLE 2:** Effect of dietary supplementation with organic acids in drinking water or in feed on cecal chyme *Salmonella* count (lg cfu/g) of broilers exposed to *Salmonella* Pullorum challenge.

**Day of age^e^**	**Unchallenge control**	**Challenge control**	**Challenge + VG^d^**	**Challenge + OA^e^ water**	**Challenge + OA^e^ feed**	**Challenge + OA^e^ water + OA^e^ feed**	***P*-value**
Cecal chyme							
7 days	4.75 ± 0.07	4.69 ± 0.06	4.7 ± 0.11	4.73 ± 0.08	4.78 ± 0.06	4.69 ± 0.10	0.100
14 days	6.48 ± 0.06^bc^	7.94 ± 0.08^a^	5.82 ± 0.07^c^	6.83 ± 0.08^ab^	6.79 ± 0.08^ab^	6.75 ± 0.07^ab^	<0.001
21 days	7.08 ± 0.12^abc^	8.32 ± 0.27^a^	5.92 ± 0.17^d^	6.93 ± 0.10^abcd^	6.90 ± 0.10^bcd^	6.85 ± 0.16^*cd*^	<0.001
42 days	8.25 ± 0.13^ab^	9.87 ± 0.08^a^	6.97 ± 0.15^d^	7.71 ± 0.12^bcd^	7.99 ± 0.38^abc^	7.11 ± 0.22^*cd*^	<0.001

*^*a*–*d*^Means within a row with no common superscript differ significantly (*n* = 8; *P* < 0.05). Data are the mean of eight replicates with 14 birds each. ^*e*^VG, virginiamycin feed grade premix. ^*f*^OA, organic acids.*

### Intestinal Histomorphology

As shown in Table 3 and Supplementary Figure S1, the *S*. Pullorum challenge increased jejunum crypt depth of birds compared with the unchallenged birds (*P* = 0.008) on day 42. Challenged birds receiving OA water, OA feed, or these in combination showed reduced jejunum crypt depth compared to the challenged control (*P* = 0.008), but showed no difference compared to the unchallenged and virginiamycin group. A greater villus height:crypt depth ratio (VH:CD ratio) was observed in challenged birds receiving OA water than those in the challenged and unchallenged group (*P* = 0.011). However, there was no significant challenge or dietary effect on the morphology of ileum of broilers on day 42 (*P* > 0.05). In addition, chicken digestive epithelial cells were not damaged by drinking acidified water.

**TABLE 3:** Effect of dietary supplementation with organic acids in drinking water or in feed on the intestinal mucosal morphology of broilers exposed to *Salmonella* Pullorum challenge at 42 days.

**Items**	**Unchallenge control**	**Challenge control**	**Challenge + VG^d^**	**Challenge + OA^e^ water**	**Challenge + OA^e^ feed**	**Challenge + OA^e^ water + OA^e^ feed**	***P*-value**
**Jejunum**							
Villus height (μm)	1129.06 ± 237.24	1079.13 ± 81.93	1172.93 ± 223.76	1197.85 ± 77.19	1026.28 ± 76.94	1078.86 ± 125.83	0.437
Crypt depth (μm)	237.04 ± 23.48^b^	293.6 ± 28.26^a^	245.32 ± 13.30^b^	221.78 ± 22.40^b^	224.76 ± 55.12^b^	242.94 ± 31.40^b^	0.008
Villus height:crypt depth ratio	4.54 ± 0.49^bc^	3.87 ± 0.31^c^	4.78 ± 0.84^ab^	5.55 ± 0.68^a^	4.37 ± 0.70^bc^	4.49 ± 0.64^bc^	0.011
**Ileum**							
Villus height (μm)	729.28 ± 81.61	704.28 ± 165.84	739.28 ± 194.71	796.85 ± 217.51	804.32 ± 97.68	748.4 ± 139.04	0.888
Crypt depth (μm)	195.3 ± 25.35	220.42 ± 45.58	208.02 ± 60.91	219.53 ± 35.15	195.57 ± 21.71	203.83 ± 32.19	0.786
Villus height:crypt depth ratio	3.77 ± 0.48	3.67 ± 0.45	3.60 ± 0.61	3.60 ± 0.57	4.15 ± 0.67	3.68 ± 0.40	0.497

*^*a*–*c*^Means within a row with no common superscript differ significantly (*n* = 8; *P* < 0.05). Data are means of eight replicates of a bird per replicate pen. ^*d*^VG, virginiamycin feed grade premix. ^*e*^OA, organic acids.*

### Metabolomic Analysis and Plasma Biochemical Parameters

**FIGURE 1:**
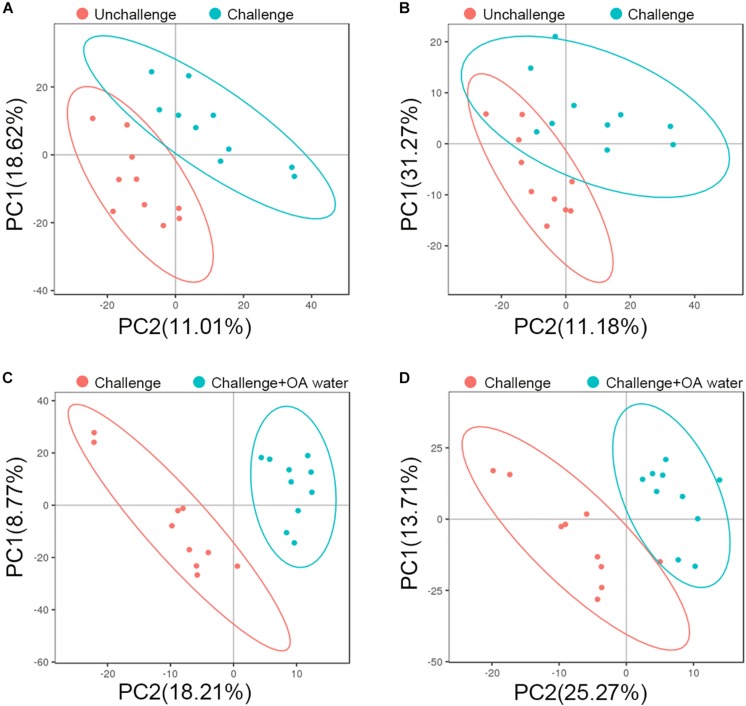
PLS-DA score plots based on data from LC-MS/MS analysis of plasma from the unchallenged control, challenged control, and the challenged group fed with organic acids in drinking water (challenge + OA water). **(A,C)** Score plots for the positive ion mode. **(B,D)** Score plots for the negative ion mode. In the score plot, each data point represents one bird plasma sample.

To characterize the metabolite changes induced by *S*. Pullorum challenge and acidified drinking water, we performed LC-MS/MS-based metabolomic analysis of plasma from the birds that were challenged, challenged and fed with acidified drinking water, and unchallenged. Although there was no distinct separation, groups did tend to cluster on the PCA score plots (Supplementary Figure S2). Using PLS-DA, we observed a clear differentiation of the challenged group and the unchallenged control in ESI + model with two components (Figure 1A) but not in ESI – model (Figure 1B). The score plots of PLS-DA also showed a distinct separation between the challenged group and the OA water group in both ESI + (Figure 1C) and ESI- models (Figure 1D).

**FIGURE 2:**
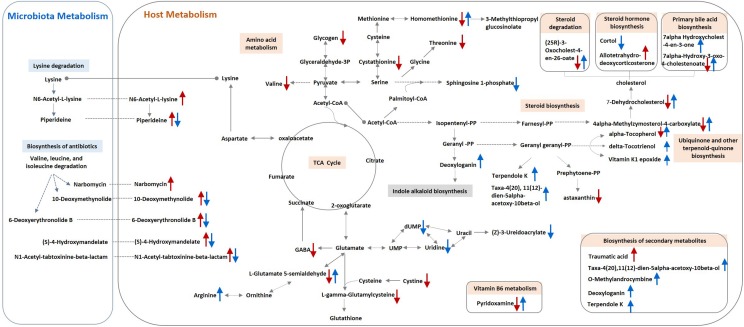
Schematic model for metabolite changes due to *S.* Pullorum challenge and organic acids intervention in broilers. A single red arrow (↑/↓) indicates increased or decreased levels of metabolites in the *S.* Pullorum challenge control compared with the unchallenged control. A single blue arrow (↑/↓) indicates increased or decreased levels of metabolites in the challenged group fed with organic acid in drinking water compared with the challenge control.

**FIGURE 3:**
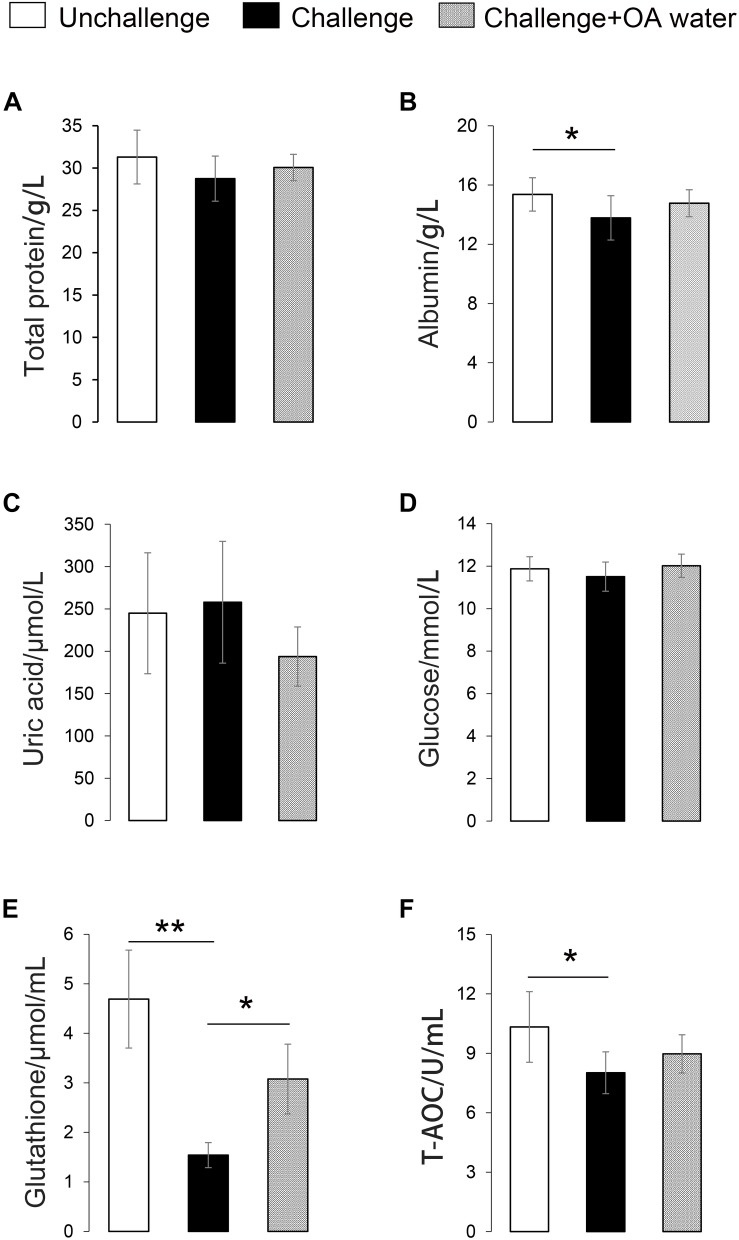
Effect of supplementation with organic acids in drinking water on plasma biochemical parameters of broilers exposed to *Salmonella* Pullorum challenge. **(A)** Total protein level. **(B)** Albumin level. **(C)** Uric acid level. **(D)** Glucose level. **(E)** Glutathione level. **(F)** T-AOC, total anti-oxidative capacity. ^∗^*P* < 0.05; ^∗∗^*P* < 0.001.

Approximately 31 differential metabolites were selected based on the criteria of VIP > 1 in PLS-DA analysis and the significant difference between the challenged group and the unchallenged control shown by the Kruskal–Wallis test (Table 4). A total of seven metabolites were involved in steroid and primary bile acid metabolism, and 10 metabolites were related to amino acid metabolism. Together with the changed levels of vitamin B6, a coenzyme for transamination reactions, these data suggested that amino acid metabolism was extensively affected by *S.* Pullorum challenge. In addition, *Salmonella* challenge caused an increase in the levels of eight potential metabolites in the plasma which involved biosynthesis of antibiotics and secondary metabolites. A total of 39 differential metabolites were identified between the challenged group and OA water group with 21 upregulated metabolites and 18 downregulated metabolites (Table 5). The metabolic pathways identified mainly involved steroid and primary bile acid metabolism, amino acid metabolism, ubiquinone and other terpenoid-quinone biosynthesis, biosynthesis of antibiotics and secondary metabolites, and pyrimidine metabolism (Figure 2). Results from the biochemical measurements showed lower plasma albumin concentration (*P* = 0.047), glutathione levels (*P* < 0.001), and T-AOC (*P* = 0.008) in the *S.* Pullorum challenged group than in the unchallenged control (Figure 3). However, challenged birds receiving OA water had a higher glutathione levels (*P* = 0.049) than those in the challenged control.

**TABLE 4:** List of the putative metabolites identified in plasma of broilers from unchallenged and *S.* Pullorum challenged control.

**Metabolites**	**Retention time**	***m/z***	**Quasi-molecular ion**	**Formula**	**Fold change^a^**	***P*-value^b^**	**VIP^c^**	**Pathway**
L-Cystathionine	0.560483333	223.0760692	M + H, M + Na	C_7_H_14_N_2_O_4_S	0.67	0.002	2.15	Glycine, serine, and threonine metabolism
L-Threonine	0.574766667	120.0655604	M + H	C_4_H_9_NO_3_	0.83	0.011	1.27	
L-Cystine	0.560483333	241.0323026	M + H	C_6_H_12_N_2_O_4_S_2_	0.75	0.015	1.69	
L-Glutamate 5-semialdehyde	0.574766667	154.048303	M + Na	C_5_H_9_NO_3_	0.61	0.009	2.31	Arginine and proline metabolism
Gamma-aminobutyric acid	0.574766667	86.06001831	M + H-H_2_O, M + H	C_4_H_9_NO_2_	0.72	0.002	1.90	Alanine, aspartate, and glutamate metabolism
L-Valine	3.441466667	100.075989	M + H-H_2_O	C_5_H_11_NO_2_	0.75	0.043	1.96	Valine, leucine, and isoleucine degradation
Gamma-L-glutamyl-L-cysteine	0.5462	289.024166	M + K	C_8_H_14_N_2_O_5_S	0.60	0.011	2.48	Glutathione metabolism
Thiourocanic acid	1.535566667	208.9778765	M + K	C_6_H_6_N_2_O_2_S	1.79	0.043	2.30	Histidine metabolism
N6-acetyl-L-lysine	0.631916667	189.1251847	M + H	C_8_H_16_N_2_O_3_	1.28	0.023	1.58	Lysine degradation
Piperideine	0.482616667	84.08092021	M + H	C_5_H_9_N	1.66	0.009	2.23	
Homomethionine	0.576166667	198.0370165	M + Cl	C_6_H_13_NO_2_S	0.52	0.005	2.17	Glucosinolate biosynthesis
Glycogen	0.619033333	701.1910202	M + Cl	C_24_H_42_O_21_	0.61	0.043	1.70	Glucagon signaling pathway
7-Dehydrocholesterol	10.14997	367.3376	M + H-H_2_O	C_27_H_44_O	0.64	0.023	2.04	Steroid biosynthesis
4Alpha-methylzymosterol-4-carboxylate	10.14997	425.3443	M + H-H_2_O	C_29_H_46_O_3_	0.70	0.035	1.75	
Lanosterol	10.29927	444.4204	M + NH_4_	C_30_H_50_O	1.13	0.043	1.01	
(25R)-3-oxocholest-4-en-26-oate	8.741267	397.3108	M + H-H_2_O	C_27_H_42_O_3_	0.64	0.035	1.57	Steroid degradation
Allotetrahydrodeoxy corticosterone	9.182733	373.2154	M + K	C_21_H_34_O_3_	1.21	0.029	1.34	Steroid hormone biosynthesis
7Alpha-hydroxy-3-oxo-4-cholestenoate	8.158333	431.3167	M + H	C_27_H_42_O_4_	0.56	0.043	2.99	Primary bile acid biosynthesis
3Alpha,7alpha,12alpha-trihydroxy-5beta-cholestan-26-al	7.2404	457.3272	M + Na	C_27_H_46_O_4_	0.79	0.043	1.19	
2-Polyprenyl-6-methoxyphenol	9.587783	299.1411	M + K	C_12_H_16_O_2_.[C_5_H_8_]_n_	0.76	0.029	1.49	Ubiquinone and other terpenoid-quinone biosynthesis
Alpha-tocopherol	10.14997	448.4177	M + NH_4_	C_29_H_50_O_2_	0.45	0.023	3.26	
Pyridoxamine	0.4969	191.0781	M + Na	C_8_H_12_N_2_O_2_	1.51	0.015	1.91	Vitamin B_6_ metabolism
6-Deoxyerythronolide B	4.238683	425.2278	M + K	C_21_H_38_O_6_	12.5	0.043	3.14	Biosynthesis of 12-, 14-, and 16-membered Macrolides
10-Deoxymethynolide	5.888833	314.2346	M + NH_4_	C_17_H_28_O_4_	4.82	0.009	2.92	
Narbomycin	10.96792	510.3421	M + H	C_28_H_47_NO_7_	1.23	0.009	1.27	
N1-acetyl-tabtoxinine-beta-lactam	0.574767	248.1248	M + NH_4_	C_9_H_14_N_2_O_5_	3.06	0.029	2.57	Monobactam biosynthesis
(S)-4-hydroxymandelate	0.939767	203.0131	M + Cl	C_8_H_8_O_4_	2.13	0.009	1.81	
N-acetylbialaphos	9.182733	383.1681	M + NH_4_	C_13_H_24_N_3_O_7_P	1.58	0.029	2.07	Biosynthesis of antibiotics
Actinamine	0.90195	189.1252	M + H-H_2_O	C_8_H_18_N_2_O_4_	1.24	0.043	1.63	
Traumatic acid	3.975083	246.1716	M + NH_4_	C_12_H_20_O_4_	3.43	0.007	2.77	Biosynthesis of secondary metabolites
Glucoselysine-6-phosphate	7.716867	427.0872	M + K	C_12_H_25_N_2_O_10_P	0.83	0.023	1.43	Phosphotransferase system

*^*a*^Fold change in the *S.* Pullorum challenged control compared with the unchallenged. ^*b*^VIP (variable importance in the projection) values were obtained from PLS-DA models. ^*c*^*P*-values were calculated from Kruskal–Wallis test.*

**TABLE 5:** List of the putative metabolites identified in plasma of broilers from *S.* Pullorum challenged group supplemented with organic acids in drinking water and challenged control.

**Metabolites**	**Retention time**	***m/z***	**Quasi-molecular ion**	**Formula**	**Fold change^a^**	***P*-value^b^**	**VIP^c^**	**Pathway**
L-Arginine	0.5462	285.09477	M + K	C_9_H_18_N_4_O_4_	1.70	0.011	3.08	Arginine and proline metabolism
L-Glutamate 5-semialdehyde	0.5747667	154.0483	M + Na	C_5_H_9_NO_3_	1.68	0.023	2.93	
L-Leucine	1.4784167	132.10267	M + H	C_6_H_13_NO_2_	0.76	0.043	2.36	Valine, leucine, and isoleucine degradation
Piperideine	0.4826167	84.08092	M + H	C_5_H_9_N	0.56	0.007	3.05	Tropane, piperidine, and pyridine alkaloid biosynthesis
Hypoxanthine	1.05125	137.04652	M + H	C_5_H_4_N_4_O	0.45	0.043	2.35	Purine metabolism
Homomethionine	0.5761667	198.03702	M + Cl	C_6_H_13_NO_2_S	1.80	0.007	2.57	Glucosinolate biosynthesis
7-Dehydrocholesterol	10.149967	367.33758	M + H-H_2_O	C_27_H_44_O	1.83	0.015	3.53	Steroid biosynthesis
4Alpha-methylzymosterol-4-carboxylate	10.149967	425.34433	M + H-H_2_O	C_29_H_46_O_3_	1.66	0.015	3.17	
Zymosterol	10.2214	385.34834	M + H	C_27_H_44_O	1.18	0.043	1.55	
(25R)-3-oxocholest-4-en-26-oate	8.7412667	397.31077	M + H-H_2_O	C_27_H_42_O_3_	3.61	0.023	4.19	Steroid degradation
7Alpha-hydroxy-3-oxo-4-cholestenoate	8.1583333	431.31674	M + H	C_27_H_42_O_4_	2.94	0.029	5.18	Primary bile acid biosynthesis
7Alpha-hydroxycholest-4-en-3-one	9.6449167	439.32085	M + H, M + K, M + Na	C_27_H_44_O_2_	1.26	0.029	2.20	
Cortol	10.755033	391.24835	M + Na	C_21_H_36_O_5_	0.80	0.043	1.67	Steroid hormone biosynthesis
Sphingosine 1-phosphate	8.4426667	380.25699	M + H, M + Na, M + H-H_2_O	C_18_H_38_NO_5_P	0.78	0.0005	2.18	Sphingolipid metabolism
Sphinganine 1-phosphate	8.6205333	382.27338	M + H	C_18_H_40_NO_5_P	0.87	0.015	1.48	
Delta-tocotrienol	8.7412667	397.31077	M + H	C_27_H_40_O_2_	3.61	0.023	4.19	Ubiquinone and other terpenoid-quinone biosynthesis
Alpha-tocopherol	10.149967	448.41767	M + NH_4_	C_29_H_50_O_2_	2.50	0.019	4.89	
Vitamin K1 epoxide	10.72645	467.35299	M + H	C_31_H_46_O_3_	1.24	0.019	2.08	
dl-Alpha-tocopherol nicotinate	9.6449167	536.40842	M + H, M + NH_4_	C_35_H_53_NO_3_	1.21	0.043	1.53	Vitamin digestion and absorption
Riboflavin	9.0477167	399.12519	M + Na	C_17_H_20_N_4_O_6_	1.14	0.029	1.41	Riboflavin metabolism
Pyridoxamine	0.4969	191.07807	M + Na	C_8_H_12_N_2_O_2_	0.63	0.015	2.72	Vitamin B6 metabolism
Ascorbate	0.6604833	159.02885	M + H-H_2_O	C_6_H_8_O_6_	0.64	0.029	1.98	Biosynthesis of phosphotransferase system (PTS)
(S)-4-hydroxymandelate	0.9397667	203.01312	M + Cl	C_8_H_8_O_4_	0.54	0.043	2.68	Monobactam biosynthesis
N1-acetyl-tabtoxinine-beta-lactam	0.5747667	248.12484	M + NH_4_	C_9_H_14_N_2_O_5_	0.28	0.007	4.17	
10-Deoxymethynolide	5.8888333	314.23462	M + NH_4_	C_17_H_28_O_4_	0.18	0.002	5.73	Biosynthesis of 12-, 14-, and 16-membered macrolides
6-Deoxyerythronolide B	4.2386833	425.22779	M + K	C_21_H_38_O_6_	0.07	0.002	4.39	
6’-Oxo-G418	3.6479167	517.24607	M + Na	C_20_H_38_N_4_O_10_	0.48	0.023	3.33	Biosynthesis of antibiotics
4-Ketocyclophosphamide	5.5045167	296.99203	M + Na	C_7_H_13_C_l__2_N_2_O_3_P	1.81	0.007	1.82	Drug metabolism—cytochrome P450
Taxa-4(20),11(12)-dien-5alpha-acetoxy-10beta-ol	7.667566667	329.2500459	M + H-H_2_O	C_22_H_34_O_3_	2.18	0.015	3.63	Biosynthesis of secondary metabolites
*O*-methylandrocymbine	0.617633333	403.2199726	M + NH_4_	C_22_H_27_NO_5_	1.60	0.043	2.94	
Deoxyloganin	8.947716667	357.1533463	M + H-H_2_O	C_17_H_26_O_9_	1.40	0.009	2.45	
Terpendole K	8.379083333	500.2800663	M + H-H_2_O	C_32_H_39_NO_5_	1.35	0.043	2.36	
(4R)-Carvone	8.6491	133.1018374	M + H-H_2_O	C_10_H_14_O	0.44	0.002	3.03	
6-Oxocineole	0.603333333	191.1047494	M + Na	C_10_H_16_O_2_	0.73	0.023	2.25	
1D-myo-inositol 1,3,4,5-tetrakisphosphate	0.525466667	482.9294166	M + H-H_2_O	C_6_H_16_O_18_P_4_	1.77	0.001	3.69	Inositol phosphate metabolism
Phytic acid	0.525466667	642.8625113	M + H-H_2_O	C_6_H_18_O_24_P_6_	1.19	0.019	1.71	
(Z)-3-Ureidoacrylate	0.88765	113.0336012	M + H-H_2_O	C_4_H_6_N_2_O_3_	0.67	0.029	2.68	Pyrimidine metabolism
Uridine	1.4098	243.0614018	M-H	C_9_H_12_N_2_O_6_	0.30	0.003	3.79	
dUMP	0.939766667	307.0337737	M-H	C_9_H_13_N_2_O_8_P	0.45	0.015	2.86	

*^*a*^Fold change in the *S.* Pullorum challenged group supplemented with organic acids in drinking water compared with the challenged control. ^*b*^VIP (variable importance in the projection) values were obtained from PLS-DA models. ^*c*^*P*-values were calculated from Kruskal–Wallis test.*

## Discussion

Young birds are highly susceptible to *S*. Pullorum colonization which results in acute clinical disease, such as a white diarrhea and high mortality, or a disease-free persistence of infection (Kogut and Arsenault, 2017). In the current study, mortality was not observed before *S*. Pullorum challenge (0–7 days of age, the first week), but occurred on 3 consecutive days in week 2 following oral challenge with 11 mortalities. This was accompanied by higher amount of *Salmonella* in cecum (*P* < 0.001) and increased litter moisture content (*t*-test, *P* = 0.028) in challenged control than unchallenged control on day 14. Further, reduced the body weight was observed at 4 days post-infection in all challenged groups except the group supplemented with virginiamycin. Similar decreased body weight at day 21 of *S*. Pullorum challenged birds, as well as growth depression by other *Salmonella* serovars, were reported previously (Wang et al., 2012; Jazi et al., 2018). However, on day 42, except for the increased FCR in the challenged control, no differences were observed in performance during the grower phase, litter moisture (Supplementary Table S4), immune organ weight ratio (Supplementary Table S5), and cecal chyme *Salmonella* count between the challenged and unchallenged control. These data may indicate an acute systemic infection in the early stages of the experiment, followed by the persistent asymptomatic infection during the grower phase (Wigley et al., 2001). By 5 weeks post-infection, it is hard to detect *S.* Pullorum from ceca contents in layer chicks, and *S.* Pullorum prefers to persist in the reproductive tract and spleen at low numbers until sexual maturity (Wigley et al., 2001). It is important to note that our *Salmonella* numeration results were not special for *Salmonella* Pullorum. Therefore, the presence of *Salmonella* Pullorum was not directly confirmed, which may be the major limitation of the current study. There was another possibility that the chicks were still exposed to environmental *Salmonella* challenge during the grower phase (Bourassa et al., 2018) although did not show any clinical signs. Supplementation of OA in the combination of feed and water could alleviate the adverse effects of *S*. Pullorum challenge on growth performance of broilers. The positive effect of OA supplementation on performance was comparable to virginiamycin in this study and to other antibiotics in a previous study (Hamid et al., 2018). The antimicrobial activity of OA and its effects on intestinal morphology could partly explain the growth improvement (Suiryanrayna and Ramana, 2015; Khan and Iqbal, 2016). And our data also showed the grower phase may be the key period for OA to exert growth-promoting effects in challenged birds.

Plasma metabolic profiling revealed a systemic metabolic perturbation caused by the *Salmonella* infection, and multiple pathways may contribute to the potential benefits of OA in *S*. Pullorum challenged birds (Figure 2). The significant changes of amino acid and steroid metabolism have been previously identified in mice during the course of *Salmonella* infection (Antunes et al., 2011; Olive and Sassetti, 2016). Chicks infected with *S.* Pullorum also had reduced levels of arginine, methionine, glycine, and tryptophan (Ross et al., 1955). *Salmonella* maintained versatile catabolic capabilities for host diverse nutrients during infection to ensure its survival and maximize parasite replication (Steeb et al., 2013). The decreased levels of glycogen, amino acids, and steroids, which could serve as carbon or nitrogen sources and supply energy, may result from the deprivation of *Salmonella* or high energy expenditure required to fight the infection (Antunes et al., 2011). These results indicate a decreased bioavailability of amino acids and other nutrients in protein and steroid biosynthesis, which could be related to the growth depression induced by *S.* Pullorum challenge. In addition, steroid biosynthesis was suppressed by *S.* Pullorum challenge on day 42, but upregulated in challenged birds by supplementation of OA in drinking water, which may be associated with host defenses against *S.* Pullorum challenge. Steroid production during inflammation is part of an important negative feedback circuit that prevents excessive host stress responses (Tait et al., 2008). Reduced levels of sphingolipid metabolites by OA were also observed. Sphingolipids have been recently reported to involve in the innate immunity against intestinal *Salmonella* infection (Huang, 2017). We therefore hypothesized that *S.* Pullorum challenge in this study triggered infection-promoting metabolic responses in the host but was suppressed by OA.

First, the stress responses mediated by steroid hormone to *Salmonella* infection could be alleviated by the addition of OA to drinking water. *Salmonella* Pullorum challenge induced an increased circulating level of allotetrahydrodeoxycorticosterone, which participates in the hypothalamic pituitary axis (HPA) in response to acute stress and acts as an allosteric modulator of the gamma amino butyric acid subunit A receptor in brain (Reddy, 2003). The other HPA-related neurosteroid, cortol (the metabolite of cortisol), which showed lower circulating levels in challenged birds supplied with acidified drinking water, may also participate in the regulation of host response to *Salmonella* challenge. The hormonal changes suggested a stress response of the host to the bacterial infection. However, *Salmonella* could benefit from the exposure to the stress hormones. Cortisol has been reported to increase intracellular *S.* Typhimurium proliferation in primary porcine alveolar macrophage cultures and in mice (Verbrugghe et al., 2011). Thus, OA suppressed the *S.* Pullorum-induced stress, and in turn, may decrease the host susceptibility to infection (Eisenreich et al., 2013).

Second, *S*. Pullorum challenge affected the levels of several metabolites related to antioxidant or immune defense, and OA seemed to restore the physiological balance by reversed modulation. This study showed that plasma levels of biological amine-containing metabolites were decreased in challenged birds, including gamma-aminobutyric acid, L-glutamate gamma-semialdehyde (intermediate metabolite in glutathione synthesis), L-cystathionine (intermediate in the L-cysteine transsulfuration pathway), and L-cystine. L-cystine is the main supplier of L-cysteine when L-cysteine production is insufficient under elevated reactive oxygen species conditions (Kodama et al., 2000; Cramer et al., 2017). Given that the importance of glutathione and cysteine in the antioxidant defense system, the reduced levels of the above-mentioned metabolites indicate a disruption of host redox homeostasis induced by *Salmonella* challenge, as reported previously (Farr and Kogoma, 1991). Additionally, the decreased levels of alpha-tocopherol and astaxanthin, and increased levels of thiourocanic acid (the derivate with immunoregulatory effects from histidine) may also indicate the infection status of challenged birds (Brummel, 1989). Results from biochemical measurements confirmed the observations obtained in metabolic profiling, as evidenced by the decreased glutathione levels and T-AOC in *Salmonella* challenge group. However, these unfavorable changes were modulated by OA supplementation, as evidenced by the increased levels of alpha-tocopherol and delta-tocotrienol, and the reduced levels of traumatic acid. Traumatic acid is a plant wound hormone belonging to the group of fatty acid derivatives and cause metabolic changes leading to adaptation to oxidative stress in human fibroblast cell (Jabłońska-Trypuć et al., 2016). An increased level of traumatin, the derivate of traumatic acid was observed in mice infected by *Toxoplasma gondii* (Zhou et al., 2018). In this study, the increased levels of traumatic acid may be a response to the disrupted redox status caused by *Salmonella* challenge but diminished by OA. Moreover, arginine is both the substrate to generate microbicidal nitric oxide radicals and the substrate *Salmonella* depend on to manifest a systemic infection (Olive and Sassetti, 2016). Further, glutamate-5-semialdehyde is a non-proteinogenic amino acid involved in the biosynthesis of arginine. The increased levels of arginine and glutamate-5-semialdehyde suggest that OA may avoid the depletion of the arginine pool induced by *Salmonella* challenge, which would further regulate cellular metabolism and function via a variety of arginine sensors (Gogoi et al., 2016).

Interestingly, a variety of putative annotated metabolites involved in antibiotics synthesis and xenobiotic degradation, and other microbiota-derived metabolites were differentially abundant, indicating a potential effect of *Salmonella* challenge and OA on the intestinal microbial community. In chicks, *Salmonella enteritidis* infection resulted in a perturbation of microbiota community, especially at the early post-infection period (Mon et al., 2015). The presumably enhanced production of antibiotics may be used by commensals or pathogens to compete and establish resilient colonization. Increased abundances of antibiotics synthesis and other secondary metabolite metabolism were reported to be associated with the dysbiosis of gut microbiota in cystic fibrosis patients, with the altered intestinal microbiota in chicken feed with antibiotics, and with low production in dairy cows (Fouhy et al., 2017; Huang et al., 2018; Mu et al., 2018). In this study, the decreased levels of metabolites involved in antibiotics synthesis and secondary metabolites are likely due to the antimicrobial activity of OA. Likewise, the OA attenuated *S*. Pullorum challenge-induced enhancement of lysine degradation, indicating that intestinal flora are probably involved in the modulation of OA, because conversion of lysine into piperidine is dependent on intestinal flora. Acidified water inhibited aerobic bacterial count and *Escherichia coli* population in cecum contents of broilers, but did not change the pH of cecum (Hamid et al., 2018). In fact, we also analyzed the cecal microbiome but failed to observe any significant change in alpha-diversity indices of cecal microbiota, differentially abundant phyla, or family across the groups on day 42. Only the abundances of *Megamonas* were significantly reduced by OA on day 42 and *Cyanobacteria* phylum on day 14 (Supplementary Figure S3). Cyanobacteria are well known for their ability to produce a wide range of toxic secondary metabolites (Tyagi et al., 1999), and *Megamonas* metabolically act as a hydrogen sink, removing the central intermediate of microbiota metabolism. Together these data suggest that the disruption of the microbiota and microbiota-derived metabolites induced by *S*. Pullorum may affect the plasma metabolome, and OA attenuate host metabolic change partly by modulating the intestinal microbiota metabolism. Further studies are required to determine the contribution of gut microbiota in host metabolism change caused by OA.

## Conclusion

Our results showed that early *S.* Pullorum challenge induced an acute systemic infection of broilers in the starter phase, followed by the grower phase without triggering clinical signs. Supplementation of OA in the combination of feed and water alleviated the adverse effects of *S.* Pullorum challenge on growth performance during the starter phase, and exerted growth-promoting effects during the grower phase. The positive effects of OA in combination, where OA in water is probably the main contributor, were comparable to virginiamycin supplementation in challenged birds. Furthermore, the abnormal levels of a variety of circulating metabolites involved in amino acid and steroid metabolism, steroid hormone biosynthesis, antioxidant and immune defense, antibiotics synthesis, and xenobiotic degradation in challenged birds were reverse modulated by OA supplementation. These data suggest that OA could moderate the systemic metabolic perturbation caused by *Salmonella* challenge, which may partly explain the potential benefits of OA in birds. Our findings contribute to a broader appreciation of the regulatory role of OA on *S.* Pullorum challenge-induced metabolic disturbances, and may provide a theoretical basis for the use of OA to reduce the effect of early pathogen exposure at the farm level.

## Data Availability Statement

The raw data supporting the conclusions of this manuscript will be made available by the authors, without undue reservation, to any qualified researcher.

## Ethics Statement

All experimental protocols were approved by the Animal Care and Use Committee of the Feed Research Institute of the Chinese Academy of Agricultural Sciences.

## Author Contributions

JW and G-HQ conceived and designed the experiments. JW analyzed the data and wrote the manuscript. DD performed animal experiments and participated in the data analysis. H-JZ, S-GW, and G-HQ supervised and provided continuous guidance for the experiment. Y-MH and Y-YW gave advice on the experimental design and manuscript revision.

## Conflict of Interest

Y-MH and Y-YW were employed by the company Trouw Nutrition R&D. The remaining authors declare that the research was conducted in the absence of any commercial or financial relationships that could be construed as a potential conflict of interest.
