# Supplementary material for: Organic Acids Modulate Systemic Metabolic Perturbation Caused by Salmonella Pullorum Challenge in Early-Stage Broilers
Source: Front Physiol. 2019 Nov 15;10:1418. doi: 10.3389/fphys.2019.01418 (PMC6873883; doi:10.3389/fphys.2019.01418)
Supplement: Supplementary file 1 [file Data_Sheet_1.docx]

Table S1. Ingredient and nutrient composition of the basal diets (air dry basis)

| Item | Starting(d 1-21) | Growing(d 22-42) |
| --- | --- | --- |
| Ingredients, % |  |  |
| Corn | 57.90 | 60.70 |
| Soybean meal | 34.85 | 30.84 |
| Soybean oil | 2.53 | 4.72 |
| Dicalcium phosphate | 2.27 | 1.82 |
| Limestone | 0.82 | 0.70 |
| Salt | 0.35 | 0.35 |
| *_DL_*-Methionine | 0.34 | 0.20 |
| *_L_*-lysine-HCl | 0.31 | 0.04 |
| Vitamin Premix^a^ | 0.02 | 0.02 |
| Mineral Premix^b^ | 0.20 | 0.20 |
| Choline chloride (50%) | 0.10 | 0.10 |
| Corn starch^c^ | 0.30 | 0.30 |
| Phytase (5,000 U/g) | 0.01 | 0.01 |
| Total | 100.00 | 100.00 |
| Nutrient levels |  |  |
| AME (MJ/kg) | 12.35 | 13.18 |
| Crude protein,% | 22.00 | 20.00 |
| Calcium,% | 1.00 | 0.90 |
| Available pppPPphosphorus,% | 0.50 | 0.40 |
| Lysine,% | 1.27 | 1.05 |
| Methionine,% | 0.55 | 0.48 |
| Methionine+cystine,% | 0.94 | 0.84 |
| Threonine,% | 0.82 | 0.69 |
| Tryptophan,% | 0.24 | 0.22 |

^a^ The vitamin premix supplied the following per kg of complete feed: vitamin A, 12 500 IU; vitamin D_3_, 2500 IU; vitamin K_3_, 2.65 mg; vitamin B_1_, 2 mg; vitamin B_2_, 6 mg; vitamin B_12_, 0.025 mg; vitamin E, 30 IU; biotin,0.0325 mg; folic acid, 1.25 mg; pantothenic acid, 12 mg; niacin, 50 mg.

^b^ The mineral premix supplied the following per kg of complete feed: Cu, 8 mg; Zn, 75 mg; Fe, 80 mg; Mn, 100 mg;I, 0.35 mg, Selenium, 0.15.

^c^ Fysal Fit 4 and virginamycin feed grade premix will substitute the respective quantity of corn starch when Fysal Fit4 and virginamycin is used, respectively.

Table S2. Drinking water pH measurement

| Treatment | Wk 1 | Wk 2 | Wk 3 | Wk 4 | Wk 5 | Wk 6 |
| --- | --- | --- | --- | --- | --- | --- |
| Normal drinking water | 7.56 | 7.74 | 7.83 | 7.59 | 7.52 | 7.44 |
| Drinking water added with organic acid blend | 3.97 | 4.12 | 4.61 | 4.34 | 4.33 | 4.36 |

Table S3. Effect of dietary supplementation with acidifiers in drinking water or in feed on growth performance of broilers (0-21 d) exposed to Salmonella pullorum challenge^1^

| Items^2^ | Unchallenge Control | Challenge  Control | Challenge  + VG^3^ | Challenge + OA Water | Challenge +  OA Feed | Challenge +  OA Water + OA Feed | *P*-value |
| --- | --- | --- | --- | --- | --- | --- | --- |
| Wk 1 |  |  |  |  |  |  |  |
| BW d 7 (g) | 196.01±9.32 | 193.59±5.18 | 201.75±7.35 | 199.48±5.49 | 195.94±7.96 | 201.21±6.09 | 0.146 |
| ADG (g) | 22.26±1.33 | 21.92±0.74 | 23.08±1.05 | 22.76±0.78 | 22.25±1.14 | 23.00±0.87 | 0.146 |
| ADFI (g) | 20.43±1.06 | 19.82±0.58 | 20.35±0.74 | 20.85±1.14 | 20.35±0.95 | 20.75±0.68 | 0.256 |
| FCR^4^ | 0.92±0.05 | 0.9±0.02 | 0.88±0.02 | 0.92±0.05 | 0.92±0.02 | 0.9±0.01 | 0.175 |
| Mortality (%) | 0.00±0.00 | 0.00±0.00 | 0.00±0.00 | 0.00±0.00 | 0.00±0.00 | 0.00±0.00 | / |
| Wk 2 |  |  |  |  |  |  |  |
| BW d 14 (g) | 482.37±17.66^a^ | 460.6±12.85^b^ | 483.15±20.14^a^ | 455.44±18.59^b^ | 459.66±21.89^b^ | 459.62±15.21^b^ | <0.01 |
| ADG (g) | 40.91±2.29^a^ | 37.81±2.04^b^ | 40.2±2.07^a^ | 36.04±2.65^b^ | 37.36±2.33^b^ | 36.13±1.72^b^ | <0.01 |
| ADFI (g) | 52.34±1.85^a^ | 47.66±2.86^b^ | 49.21±1.81^b^ | 46.87±2.61^b^ | 47.17±2.2^b^ | 47.87±1.98^b^ | <0.01 |
| FCR | 1.27±0.05^b^ | 1.26±0.03^b^ | 1.23±0.03^c^ | 1.3±0.03^a^ | 1.26±0.03^b^ | 1.31±0.02^a^ | <0.01 |
| Mortality (%) | 0.00±0.00 | 1.79±3.31 | 0.00±0.00 | 2.68±3.70 | 1.79±3.31 | 3.57±3.82 | 0.105 |
| Wk 3 |  |  |  |  |  |  |  |
| BW d 21 (g) | 928.45±34.81^a^ | 876.7±42.25^b^ | 933.25±41.88^a^ | 891.33±28.93^ab^ | 901.1±19.35^ab^ | 918.36±29.5^ab^ | 0.027 |
| ADG (g) | 61.03±4.52 | 58.87±4.69 | 63.91±3.18 | 60.31±3.07 | 60.47±4.03 | 61.94±4.19 | 0.222 |
| ADFI (g) | 92.2±4.16 | 89.36±5.13 | 89.43±3.11 | 88.7±4.02 | 87.21±4.35 | 90.91±4.55 | 0.270 |
| FCR | 1.52±0.09^a^ | 1.52±0.09^a^ | 1.4±0.04^b^ | 1.47±0.05^ab^ | 1.45±0.09^ab^ | 1.47±0.04^ab^ | 0.011 |
| Mortality (%) | 0.00±0.00 | 1.04±2.95 | 1.04±2.95 | 0.00±0.00 | 1.04±2.95 | 1.14±3.21 | 0.846 |

Means within a row with no common superscript differ significantly (*n* = 8; *P* < 0.05), data are the mean of 8 replicates with fourteen birds each. The same as below.

VG, Virginamycin feed grade premix; OA, organic acids; FCR, feed conversion ratio (feed:gain, g:g).

^1^Birds were challenged with S. enteric subsp. *Pullorum* on d 8-10.

.

Table S4. Effect of dietary supplementation with acidifiers in drinking water or in feed on litter moisture content (%) and litter scores in broilers^1^

| Day of age | Unchallenge Control | Challenge  Control | Challenge  + VG^3^ | Challenge + OA Water | Challenge +  OA Feed | Challenge +  OA Water + OA Feed | *P*-value |
| --- | --- | --- | --- | --- | --- | --- | --- |
| litter moisture content | | | | | | | |
| 21 d | 74.40±1.07 | 75.42±0.52 | 74.44±0.80 | 74.85±0.7 | 75.21±0.72 | 74.57±0.81 | 0.059 |
| 42 d | 75.65±3.17 | 78.22±1.9 | 76.33±2.18 | 76.56±2.47 | 76.58±1.19 | 76.42±1.32 | 0.299 |

Data are means of 8 replicates.

VG, Virginamycin feed grade premix; OA, organic acids.

^1^Birds were challenged with S. enteric subsp. *Pullorum* on d 8-10.

Table S5. Effect of dietary supplementation with acidifiers in drinking water or in feed on immune organ weight ratio (%) of broilers^1^ at 42 d

| Items^2^ | Treatment 1 (Negative Control) | Treatment 2 (Positive Control) | Treatment 3  (AGP) | Treatment 4  (Selko pH) | Treatment 5  (Fysal Fit 4) | Treatment 6  (Selko pH + Fysal Fit 4) | *P*-value |
| --- | --- | --- | --- | --- | --- | --- | --- |
| Thymus weight ratio | 1.59±0.34 | 1.15±0.35 | 1.26±0.47 | 1.37±0.41 | 1.36±0.26 | 1.59±0.75 | 0.332 |
| Spleen weight ratio | 1.34±0.28 | 1.21±0.23 | 1.16±0.28 | 1.18±0.19 | 1.02±0.15 | 1.37±0.34 | 0.107 |
| Bursa weight ratio | 1.54±0.44 | 1.29±0.23 | 1.2±0.49 | 1.45±0.48 | 1.43±0.4 | 1.32±0.38 | 0.651 |

Data are means of 8 replicates of a bird per replicate pen

VG, Virginamycin feed grade premix; OA, organic acids.

^1^Birds were challenged with S. enteric subsp. *Pullorum* on d 8-10.

^2^Thymus, bursa and spleen weight ratios are expressed as a percentage of body weight.


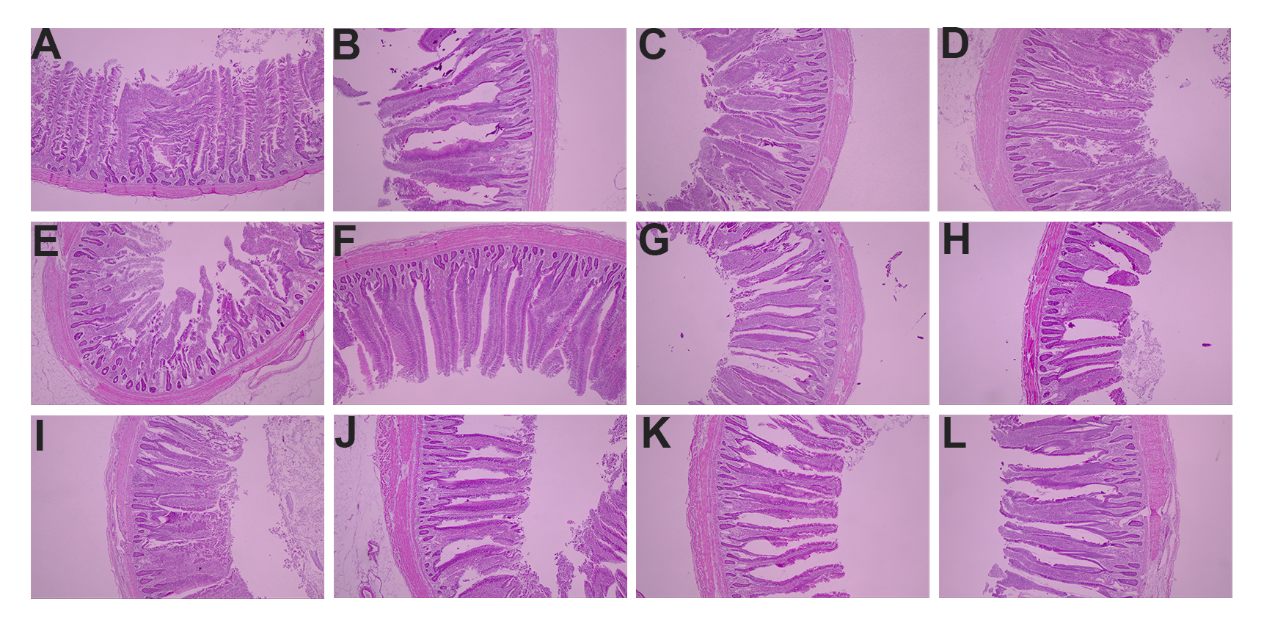


Figure S1. Hematoxylin and eosin-stained images of intestinal morphological structure in broilers on 42d (40 × magnification). A-F, from jejunum samples; G-L from ileum samples. A, G, from unchallenge control; B, H, from challenge control; C, I from challenge + virginamycin; D, J, from challenge + OA water; E, K, from challenge + OA feed;

F, L, from challenge + OA water + OA feed.


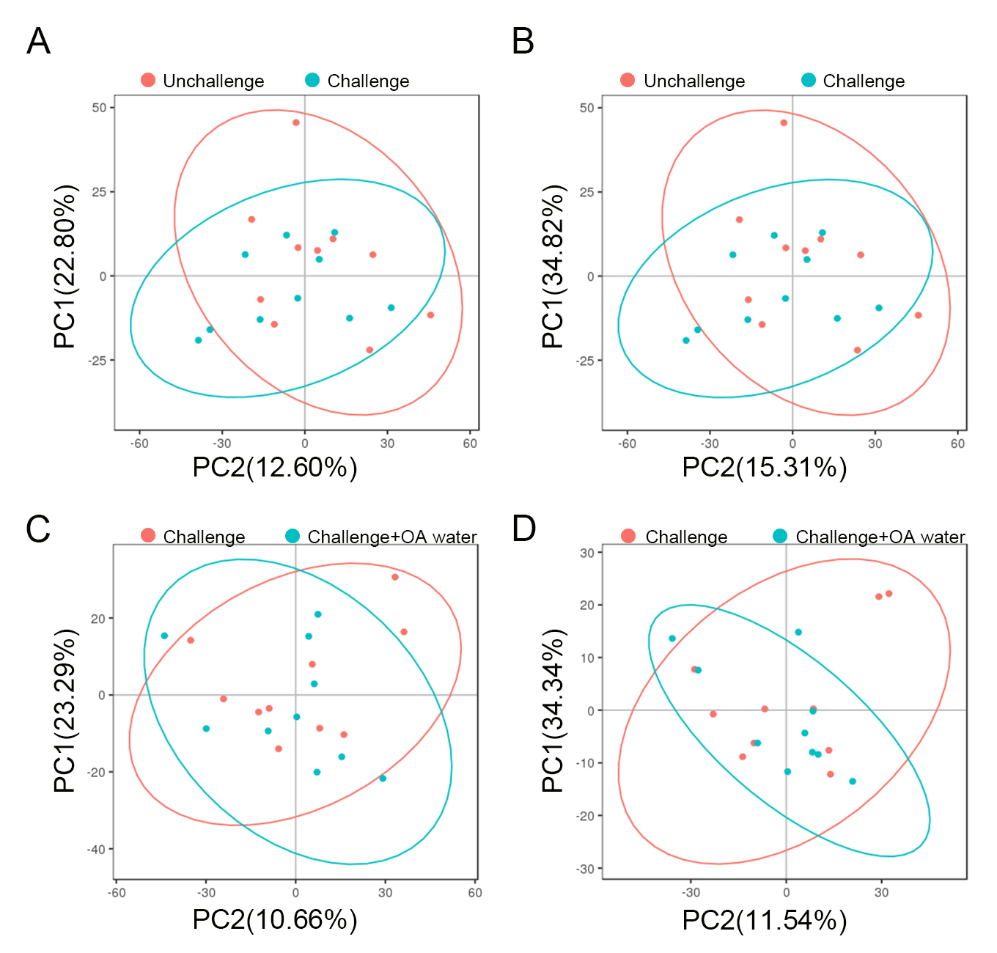


Figure S2. PCA plots based on data from LC-MS/MS analysis of plasma from the unchallenged control, challenged control, and the challenged group fed with organic acid in drinking water (challenge + OA water). (A, C) score plots for the positive ion mode, (B, D) score plots for the negative ion mode. In the score plot, each data point represents one bird plasma sample.


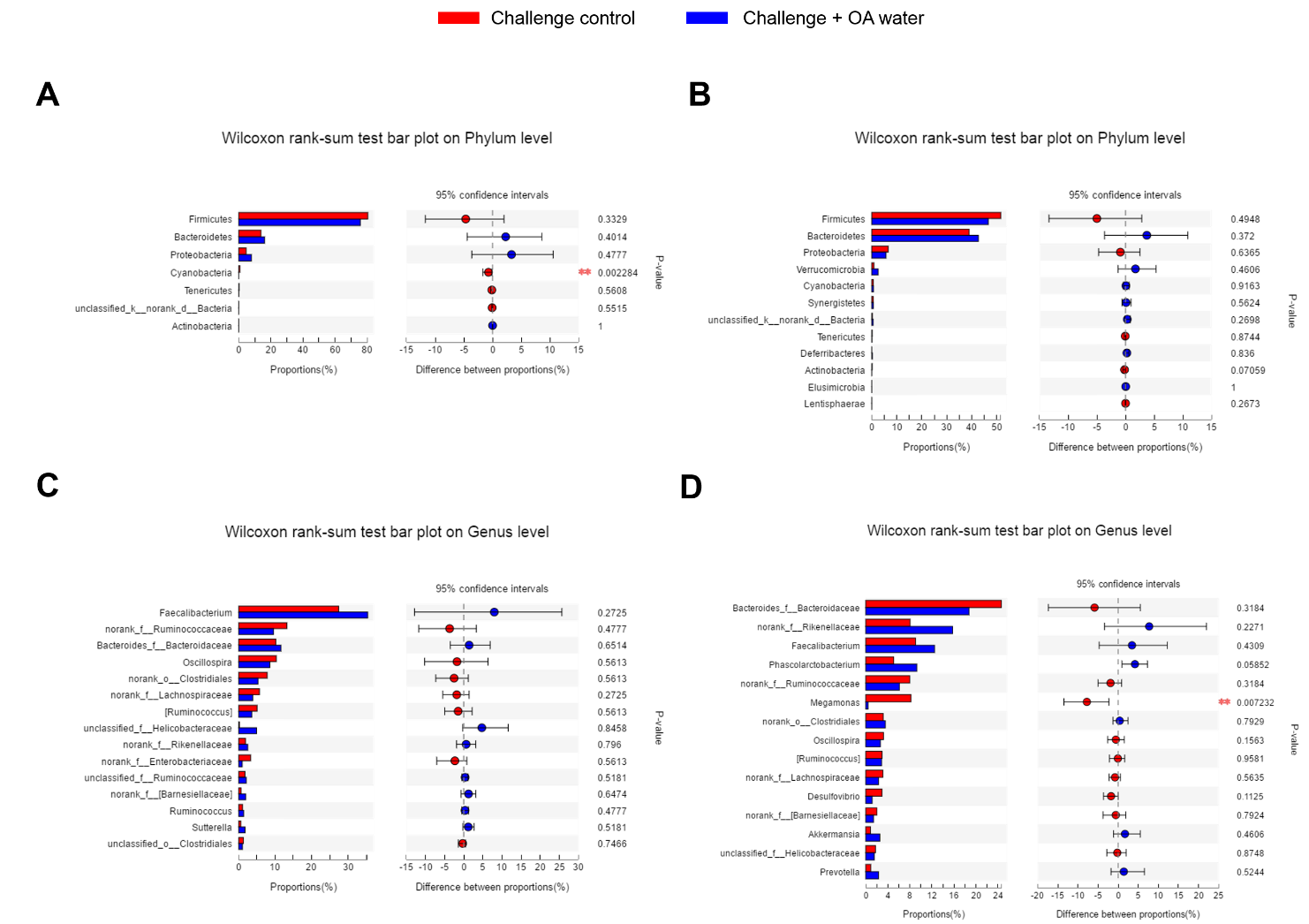


Figure S3. Differentially abundant phylum and genra in cecal chyme from broilers on d 14 (A, C) and d 42 (B, D). Birds were challenged with S. enteric subsp. *Pullorum* on d 8-10. Challenge control, S. pullorum-challenged group with the basal diets; Challenge + OA water, the challenged group receiving the control diets and the drinking water added with organic acid blend.
